# Ventrolateral prefrontal-amygdala repetitive transcranial magnetic stimulation (rTMS) modulation of impulsivity in borderline personality disorder: a proof-of-concept study

**DOI:** 10.1038/s41598-026-56692-2

**Published:** 2026-06-17

**Authors:** Reza Tadayonnejad, Rani Gera, Stephanie A. Chu, Hope Hawkins, Emmily Hovhannisyan, Thuc Doan P. Ngo, Juliana Corlier, Andrew F. Leuchter

**Affiliations:** 1 TMS Clinical and Research Service, Neuromodulation Division, UCLA Semel Institute for Neuroscience and Human Behavior, 760 Westwood Plaza, Room # 57.464, Los Angeles, CA 90024 USA; 2 Department of Psychiatry & Biobehavioral Sciences, David Geffen School of Medicine at University of California, Los Angeles, CA USA; 3 Division of the Humanities and Social Sciences, California Institute of Technology, Pasadena, CA USA

**Keywords:** Borderline personality disorder, Ventrolateral prefrontal-amygdala circuit, Impulsivity, Transcranial magnetic stimulation, Diseases, Neuroscience, Psychology, Psychology

## Abstract

Impulsivity and emotional dysregulation are core symptoms of Borderline Personality Disorder (BPD) for which therapeutic options are very limited. Prior neuroimaging studies suggest that BPD symptoms may be related to fronto-amygdala circuit dysfunction. This proof-of-concept study examined whether individualized ventrolateral prefrontal cortex (VLPFC)-amygdala circuit modulation with Repetitive Transcranial Magnetic Stimulation (rTMS) could alter impulsivity and emotional dysregulation in BPD. Eleven participants with BPD underwent resting-state functional Magnetic Resonance Imaging (rsfMRI) scans to identify individualized right and left VLPFC targets with the strongest functional connectivity to the ipsilateral amygdala. They then performed delayed discounting and emotional reactivity to social exclusion tasks to assess impulsivity and emotional reactivity, respectively, followed by a single session of intermittent Theta Burst Stimulation (iTBS) administered to left and/or right VLPFC targets. Tasks were repeated after stimulation to assess change in impulsivity and reactivity. Mixed-model linear regression analysis showed that right VLPFC, but not the left or bilateral VLPFC stimulation, was associated with a significant reduction of impulsivity (χ2(2) = 7.76, *p* = 0.02) but not emotional reactivity. No subjects experienced significant adverse events. These results suggest that the right VLPFC iTBS is a safe, tolerable, and promising procedure for modulating impulsivity in BPD.

**Supplementary Information:**

The online version contains supplementary material available at 10.1038/s41598-026-56692-2.

## Introduction

Borderline Personality Disorder (BPD) is a complex psychiatric condition affecting 0.7–2.7% of the adult population, ^1,2^, and is characterized by marked impulsivity, pervasive and persistent patterns of unstable affect and interpersonal relationships, and extreme emotional reactivity.^3^ BPD imposes a high burden on mental health systems, accounting for 10% of outpatient psychiatric visits and up to 20% of inpatient admissions^4–6^. Psychotherapy is the first-line treatment for BPD with modest effect^7,8^ with pharmacology recommended only as an adjunctive approach for targeted management of discrete comorbidities (e.g., depression)^9^. There are currently no approved biological or brain-based treatments for addressing the core features of BPD.

Functional and structural imaging studies have shown frontolimbic network dysfunction in BPD, and symptoms have been hypothesized to arise from hyperactivation/hyperreactivity of limbic structures. In particular, studies have demonstrated increased amygdala and insula reactivity in conjunction with impaired top-down regulation from cortical regions (e.g., prefrontal cortex, anterior cingulate cortex) ^10–14^.

Advances in neuromodulation technologies, particularly neuroimage-guided non-invasive brain stimulation techniques such as Repetitive Transcranial Magnetic Stimulation (rTMS), provide the opportunities to target and modulate specific brain circuits in an individualized manner. Several recent studies have examined the efficacy of rTMS in BPD applied to targets that are commonly used in treatment of depression, such as the dorsolateral prefrontal cortex (DLPFC) or dorsomedial prefrontal cortex (DMPFC). In one study with a small sample size of 14 patients with BPD^15^, 5 Hz rTMS over the DMPFC resulted in a significant reduction in Clinical Global Impression Scale for BPD (CGI-BPD)^16^, and comorbid anxiety and depression symptoms in the active compared to the sham group. However, no significant change was detected between groups in Borderline Symptoms List (BSL)^17^ or Barratt’s Impulsivity Scale (BIS)^18^ measures. In another study of BPD patients with comorbid Major Depressive Disorder (MDD), 20 Hz rTMS over the DMPFC was found to be effective in reducing depression comorbidity but not impulsivity and core BPD pathology^19^. The effect of 1 Hz vs 5 Hz rTMS (no sham), respectively, over the right vs left DLPFC was tested in another BPD study^19^. Authors reported that both protocols resulted in a significant reduction in CGI-BPD. Lastly, a recent systematic review and network meta-analysis reported no significant effect of rTMS (administered at frequencies of 1, 5,10 or 20 Hz) for reducing core BPD symptom severity^20^.

One particularly promising frontolimbic but untested stimulation target is the ventrolateral prefrontal-amygdala (VLPFC-amygdala) circuit. The most consistent neuroimaging finding in BPD is amygdala hyperactivity,^21,22^ which has been suggested as a potential predictor of treatment response^23^ and therapeutic target for neurofeedback in BPD.^24^ VLPFC has been shown to have direct structural and functional connectivity with the amygdala,^25,26^ and stimulation of this region has been shown to modulate amygdala regional activity.^27,28^.

In this proof-of-concept study, we examined whether neuroimage-guided rTMS applied to a VLPFC-amygdala circuit could modulate some of the core symptoms of BPD. We hypothesized that rTMS administered to VLPFC-amygdala targets would be safe, tolerable, and could modulate impulsivity and emotional hyperreactivity in BPD.

## Materials and methods

### Study design and participants

**Fig. 1:**
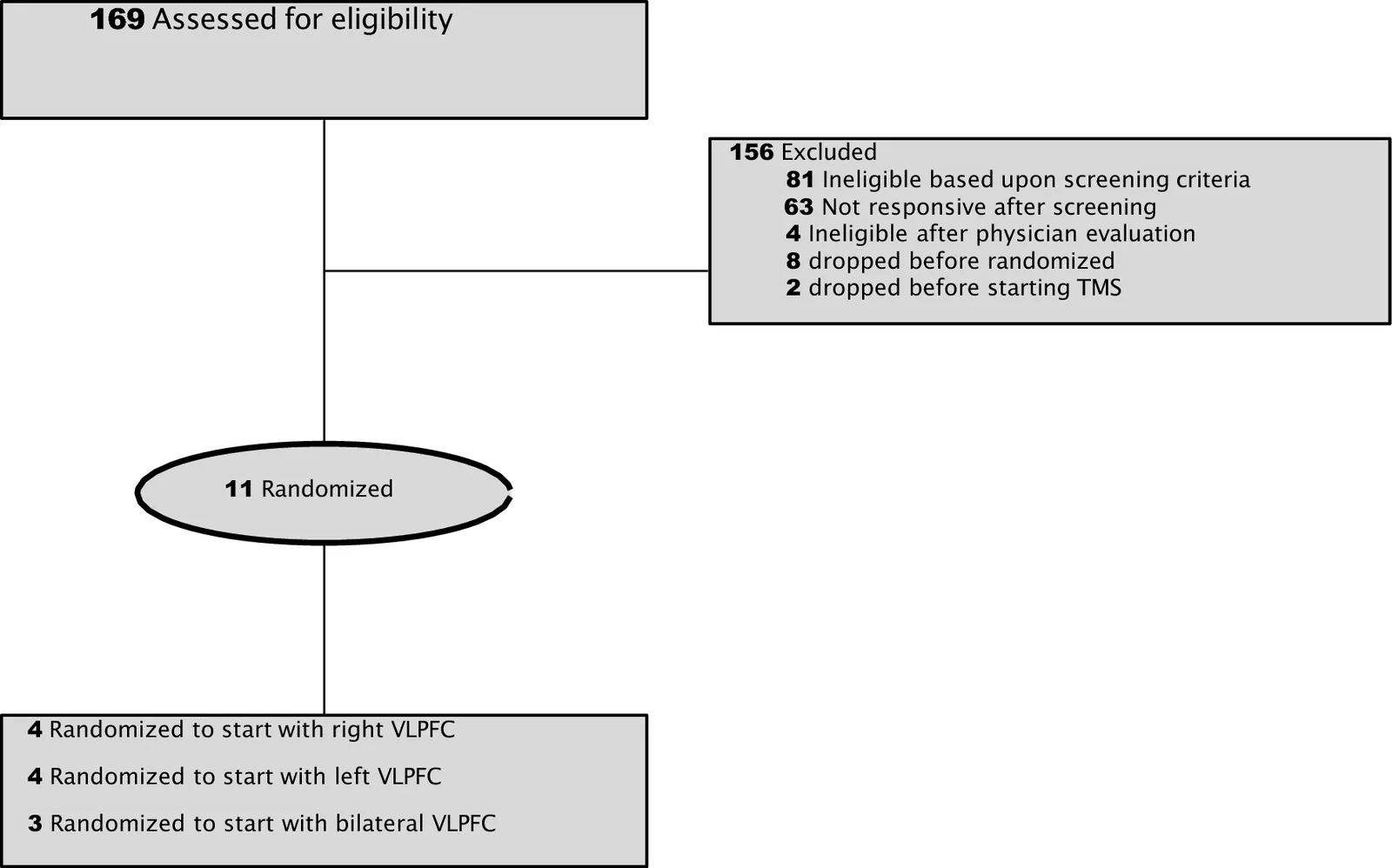
Study Flow Diagram. Following screening, enrollment, and fMRI scanning to identify VLPFC targets, subjects were assigned to receive left, right, and bilateral VLPFC rTMS in random and counterbalanced order.

Subjects with BPD were recruited using community advertisement, with BPD diagnosis established with a diagnostic interview and administration of the Zanarini Rating Scale^29^. Subjects with comorbid Axis 1 bipolar, psychotic, or acute substance use disorders were excluded based upon the Mini International Neuropsychiatric Interview (MINI).^30^ Subjects with comorbid depression or anxiety disorders were not excluded given the high prevalence of depression and anxiety condition in BPD. Informed consent was obtained from each subject before participating in this study. All procedures were approved by the UCLA Institutional Review Board (UCLA IRB ID: IRB-23-1207, approval date:1/5/2024, study start date including participant recruitment:1/19/2024). All methods and experiments were performed in accordance with UCLA IRB. This study was registered retrospectively with ClinicalTrials.gov (date of registration: 10/31/2025, ClinicalTrials.gov Identifier# NCT07223619). Please see Fig. 1 for an overview of the study flow.

The experimental paradigm included performing initial eligibility screening which was conducted for a total of 169 subjects. 85 out of 169 subjects were found ineligible based on initial or physician screening. However, more than 70 subjects were non-responsive after initial screening. We speculate that the non-treatment nature of our study, particularly for this patient population, could have a role. Finally, eleven subjects (10 female/1 male, average age: 27.1 ± 8.8 years, Table 1) were eventually enrolled for this study. Subjects were first interviewed by a board-certified psychiatrist (RT) for establishing BPD and other comorbid diagnoses. Each subject participate in four visits. The MRI scanning (see below) was done during the first visit. In the second, third and fourth visit, subjects performed two tasks (delayed discounting and emotional reactivity) immediately before and after stimulation of the right, left or bilateral VLPFC targets (administered in a randomized order).

**Table 1:** Demographic and clinical information of the study participants.

Subject ID	Age	Sex	Comorbidity
#1	24	F	MDD, GAD
#2	23	F	GAD, ADHD
#3	24	F	MDD
#4	33	M	MDD, GAD
#5	26	F	MDD, GAD
#6	26	F	MDD, PTSD, GAD
#7	22	F	MDD
#8	50	F	MDD
#9	20	F	GAD
#10	20	F	None
#11	31	F	MDD, GAD

None of the participants was on psychotropic medication.

MDD: Major Depressive Disorder; GAD: Generalized Anxiety Disorder; ADHD: Attention-Deficit/Hyperactivity Disorder; PTSD: Post-Traumatic Stress Disorder.

### Neuroimaging

Structural (high-resolution T1-weighted) and resting-state functional Magnetic Resonance Imaging (rsfMRI) scans were acquired on a Siemens Prisma 3 Tesla scanner. Structural images were collected using a volumetric T1-weighted MPRAGE sequence (repetition time (TR) = 2500 ms, echo time (TE) = 2.29 ms, inversion time (TI) = 900 ms, flip angle = 8°, field of view (FOV) = 240 mm, 160 slices, acquisition matrix = 192 × 192, 7 min). rsfMRI data were acquired using an echo-planar imaging (EPI) T2*-weighted gradient echo sequence (TR = 800 ms, TE = 36.6 ms, flip angle = 52°, voxel size = 2 mm, FOV = 210 mm, acquisition matrix = 64 × 64, 10 min per run). Three runs of rsfMRI were acquired; two were acquired with anterior-to-posterior (AP) phase encoding, and one was acquired with posterior-to-anterior (PA) phase encoding. fMRIPrep v20.2.6 was used to preprocess all structural and functional MRI data^31^.

Anatomic preprocessing consisted of skull stripping and intensity non-uniformity correction with Advanced Normalization Tools (ANTs), using the OASIS30ANTs target template. FSL FAST was used to segment brain tissue into CSF, white matter, and gray matter. Non-linear registration and ANTs volume-based spatial normalization to MNI standard space were performed. For each functional imaging run, fMRIPrep generated a reference volume and skull-stripped version. FSL FLIRT was used to run co-registration to a T1w reference. FSL mcflirt was used for head-motion parameter estimation. Slice time correction was performed with AFNI. The image was then resampled to MNI standard space. ICA-AROMA motion artifact removal using the “non-aggressive” denoising option was performed after spatial smoothing with an isotropic, Gaussian kernel of 6 mm FWHM. Confounds were then calculated (framewise displacement (FD), DVARS, and three region-wise global signals). Extraction of physiological regressions for component-based noise correction (CompCor) was performed.

**Fig. 2:**
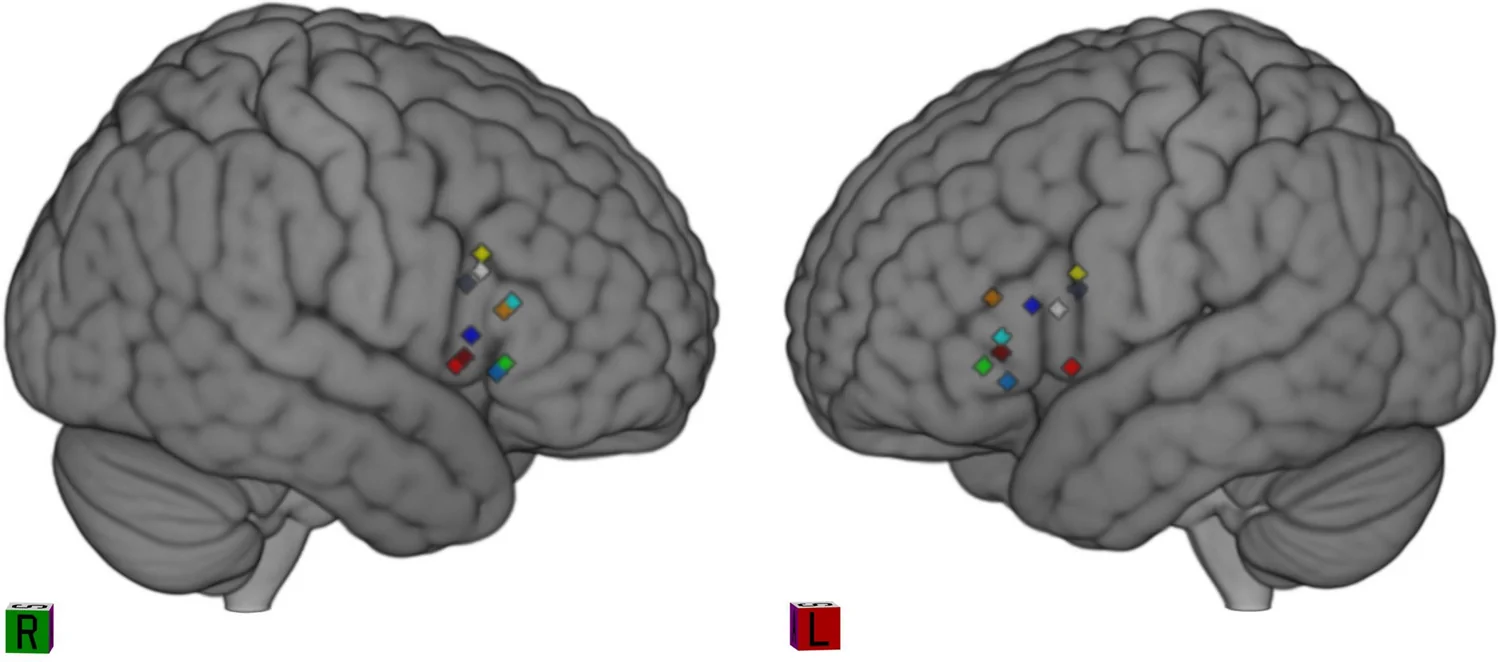
Individualized neuronavigation targets are localized in the right and left ventrolateral prefrontal cortex based on having the highest connectivity with the right and left basolateral amygdala seeds. Each subject’s left and right targets are indicated by an individual color for that subject.

To determine each patient’s individualized VLPFC targets, the functional connectivity between the patient’s basolateral amygdala (BLA) and VLPFC was performed for each hemisphere (left and right). The left and right basolateral amygdala seed masks used in this study were defined in prior literature using a histological and probabilistic atlas^27,28^. The VLPFC (e.g., inferior frontal gyrus [IFG]) masks were created using the Harvard–Oxford cortical atlas, then masked with an FSL-derived gray matter probability mask (FSL v6.0) thresholded at a 50% probability to constrain the search area to gray matter only. The resulting VLPFC – BLA functional connectivity maps were thresholded at the voxel level at *p* < 0.05 uncorrected. From these functional connectivity maps, the coordinates with the peak z-scores within the left and right VLPFC were chosen to be the main stimulation targets. Backup targets were chosen to be the most posterior VLPFC location possible within the connectivity map to be used in case the patient could not tolerate stimulation at the main target (Fig. 2).

### rTMS methods

Prior to rTMS stimulation, each subject’s resting motor threshold (MT) was determined using procedures previously described^32^ and electromyogram (EMG) monitoring of the interosseous muscle of the hand with the Brainsight system (Rogue Research, Montreal, Canada). 1800 pulses of intermittent Theta Burst Stimulation (iTBS, 3-pulse bursts [at 50 Hz] in 2-s “trains” followed by an 8 s intertrain interval) were administered at 120% MT in a single nine-minute session to the left, right, or sequentially to bilateral VLPFC targets with the Magventure R30 system (MagVenture, Farum, Denmark) equipped with the B70 coil, with placement guided with the Brainsight system. Order of stimulation was randomly assigned and counterbalanced across participants. In subjects who could not tolerate stimulation over the primary targets because of discomfort, the backup targets (which were more slightly posterior but still with strong amygdala functional connectivity) were used. Eight of the 11 subjects tolerated stimulation at 120% MT at the primary target; in 3 subjects the backup targets were used with 100–110% MT intensity. Few subjects reported mild discomfort/pain during stimulation session. No subject dropped from the study due to side effects.

### Tasks

Participants came for three rTMS visits with 3–5 days intervals between each visit, with delayed discounting and emotional reactivity tasks performed immediately *before* and *after* the right, left, and bilateral VLPFC rTMS. The 3–5 days interval between sessions exceeds the known duration of carryover effects of single iTBS sessions, which typically last 20 min to 1 hour^33^.

#### Delayed discounting task

Participants completed a 36-trial intertemporal choice task, adapted from Figner et al.,^34^ before and after each VLPFC stimulation session. In each trial, they made a binary decision between a sooner-smaller (SS) and a later-larger (LL) monetary reward. The SS amount, expressed in US dollars, was pseudo-randomly drawn from a normal distribution (mean = 15; standard deviation = 5), capped between 5 and 28.33, and then jittered by 2 (actual SS range was $3.4–$24.5). The LL reward was set to be either 0.5%, 1%, 10%, 15%, 20%, 25%, 30%, 50%, or 75% larger than the SS reward. The systematic manipulation of relative magnitudes was critical to varying choice conflict. Critically, this design includes trials with very small (0.5%, 1%) or very large (50%, 75%) differences which are expected to produce low conflict, as most participants would strongly favor one of the options. In contrast, moderate differences (10%, 15%, 20%, 25%, 30%) were assumed to generate higher conflict, requiring greater self-control to resist the sooner, yet smaller, reward. The SS reward was offered either today (“now”; 18 trials) or in two weeks (“not now”; 18 trials), with the LL reward scheduled for delivery either two or four weeks following the SS option.

To indicate their choice, participants clicked on a radio button located below their preferred option. Choices were self-paced. Six sequences were drawn (one per session) and were counterbalanced across stimulation targets. The trial order was randomized for each participant, and all participants completed the same set of choices. The task took around 3 min (mean: 185.17 s). At the end of each day, a lottery was conducted, where one trial per day was randomly selected from the two daily sessions, and the chosen option was paid according to the amount and time of payment. If the selected choice was an immediate reward, participants received this additional payment right away. If the selected choice was a delayed reward, payment was made at the corresponding delay by two weeks. The task, adapted from the Experiment Factory, was programmed in JavaScript^35^, primarily using the jsPsych library^36^.

#### Emotional reactivity task

For the emotional reactivity task, subjects viewed images with a social isolation theme (i.e., showing one individual who was rejected or excluded from interaction with others). Images were selected de novo or from an established and previously published dataset ^37^. Six blocks (each containing 14 photos) were presented before and after right, left, or bilateral VLPFC stimulation. Blocks had a collection of photos with similar complexity and valance distribution, and no photos were repeated across experiments for a subject. After each photo presentation, subjects were asked to rate how much that photo-induced “distressed feeling” (negativity rating) from 1 (no distress at all) to 10 (maximum distressed feeling).

### Statistical analysis

Data preprocessing and statistical modeling were conducted in R (https://www.r-project.org) and Python ^38^. Mixed-effects linear and logistic regression models were fitted using the lme4 package^39^ in R.

#### Intertemporal choice (delay discounting) task analysis

To assess the impact of VLPFC stimulation on intertemporal choices, as a behavioral proxy for impulsivity favoring sooner, smaller rewards, we fit a mixed-model logistic regression model predicting each trial’s choice (SS vs. LL). The main independent variables were stimulation type (Right, Left, or Bilateral), time (pre- vs. post-stimulation), and their interaction, which served as the main indicator of stimulation-induced behavior change. We controlled for reward magnitude by including the SS amount and the relative difference between SS and LL (i.e., the sum ratio, LL/SS). We also included trial type (Now vs. Not-Now) and time difference (2 vs. 4 weeks) to account for the temporal parameters of each option. To account for potential (and expected) non-linear effects of intermediate sum ratio values, we added a dummy variable indicating trials within the moderate conflict range (10–30% difference), its interactions with stimulation type and time were also included. We specified random intercepts for patients. The model in *lmer* syntax^39^ was formulated as follows:

$$\begin{gathered} Choice \, \sim \, Stimulation \, type \, \times \, Time \, \times \, High \, choice \, conflict \, indicator \, \hfill \\ + \, SS \, amount \, + \, Sum \, ratio \, + \, Trial \, type \, \hfill \\ + \, Payment \, time \, difference \, + \, \left( {1| \, Patient} \right) \hfill \\ \end{gathered}$$

This comprehensive model allowed us to control for all relevant variables and test whether different VLPFC stimulation conditions differentially affect intertemporal choices in a single, unified analysis. We evaluated the main effects and interactions using a Type II Wald chi-square analysis implemented in the car package^40^. To focus on the simple effects of interest, we set Right stimulation and the Low image arousal level as baseline levels. We then re-ran the model with Left and Bilateral Stimulation type as the baseline levels to test whether they showed a significant change following stimulation and whether there was a difference between the two. To verify that session order did not influence our findings, we repeated our analysis while including session number (1st, 2nd, or 3rd visit) as a covariate. For completeness, we conducted two additional analyses: one in which we omitted the High-Choice Conflict indicator and re-ran the analysis on the entire dataset, and another in which we restricted the analysis to only high-conflict choices. Finally, to ensure results were not driven by outliers or individual participants, we conducted leave-one-out sensitivity analyses by systematically excluding each participant and re-running the model. We report the p-values for the Stimulation Type × Time interaction across all iterations for both the full dataset and the analysis restricted to high-conflict trials.

#### Emotional reactivity analysis

To examine the effects of the VLPFC stimulation target on negativity ratings in response to social isolation images, we employed a mixed-effects linear regression. We used negativity ratings as the dependent variable. Stimulation type (Right, Left, or Bilateral), time (pre- vs. post-stimulation), and image arousal category (low vs. high), along with all their interactions, were included as independent variables. Random intercepts were specified for patients. The model in *lmer* syntax was formulated as follows:

$$\begin{gathered} Image \, negativity \, rating \, \sim \, Stimulation \, type \, \times \hfill \\ \, Time \, \times \, Image \, arousal \, level \, + \, \left( {1| \, Patient} \right) \hfill \\ \end{gathered}$$

Our main goal was testing whether different VLPFC stimulation targets differentially influenced negativity ratings (as indicated by a Time × Stimulation interaction), potentially as a function of image arousal level (as reflected in a Time × Stimulation × Arousal Category interaction). Main effects and interactions were evaluated using a Type II Wald chi-square analysis To focus on the simple effects of interest, we set the Right, Left or Bilateral stimulation and the critical sum ratio range (marked by the High choice conflict indicator) as baseline levels. We then re-ran the model with other Stimulation types as the baseline levels to test whether they showed a significant change following stimulation and whether there was a difference between the different conditions. To verify that session order did not influence our results, we repeated our analysis while including session number (1st, 2nd, or 3rd visit) as a covariate.

## Results

### Delay discounting task performance

**Fig. 3:**
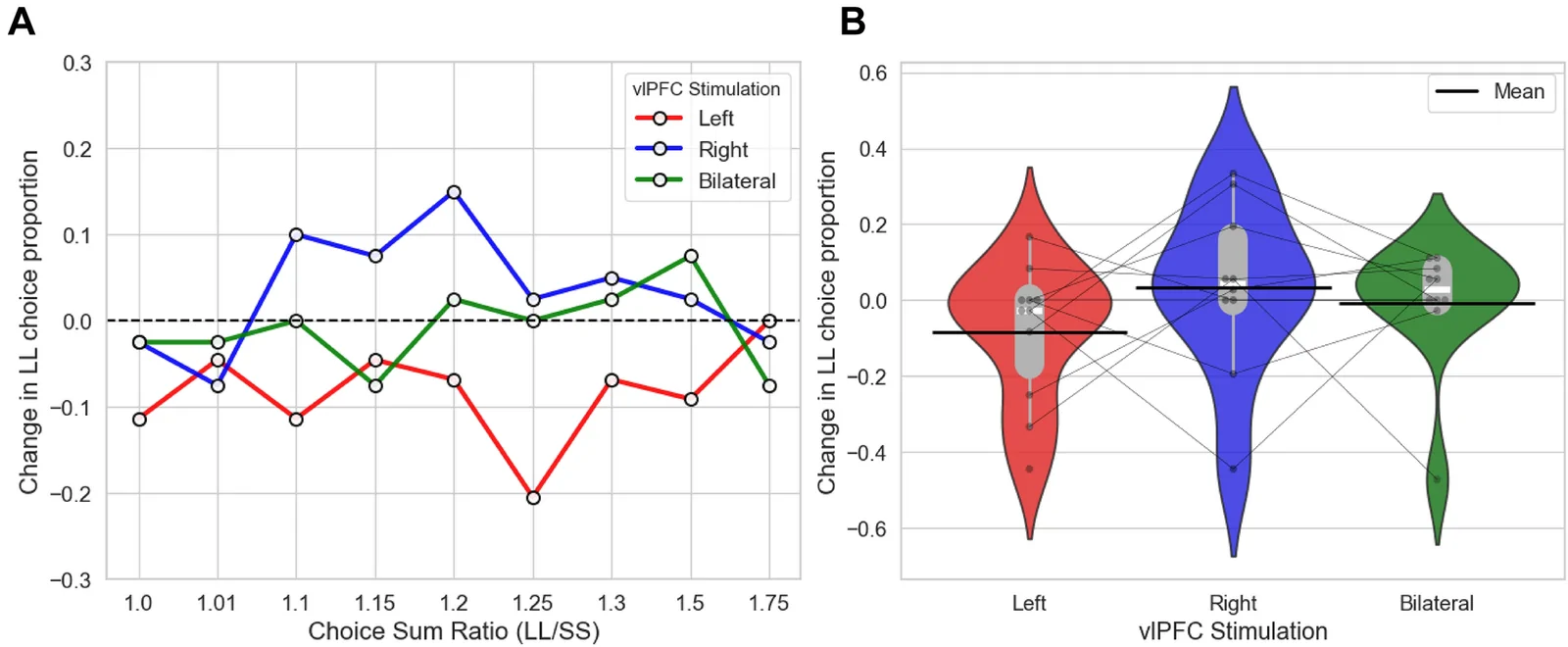
Change in intertemporal choices. Change in proportion of Later-Larger (LL) over Sooner-Smaller (SS) choices following stimulation of each target (post minus pre stimulation). Higher (more positive) scores represent lower delay discounting, aligned with decreased impulsivity. Lower (more negative) scores were aligned with increased impulsivity. (**A**) Changes across different LL/SS sum ratios. In the higher-conflict range (1.1–1.3), LL choices increased following right and decreased following left VLPFC stimulation. Points represent the mean. (**B**) Aggregated differences across all sum ratio levels. Violin plots, boxplots, and mean values are shown. Individual participant data points are connected by lines to illustrate within-subject changes.

Mixed-models logistic regression model showed a differential effect of the VLPFC stimulation target on choices, manifested as a significant *Stimulation Type* × *Time* interaction effect (χ^2^_2_ = 11.23, *p* = 0.004; Table S1). Right VLPFC stimulation was associated with a significant increase in LL choices relative to left VLPFC condition (which showed a numeric decrease) and bilateral stimulation (which showed little numeric change) (Fig. 3). This was confirmed by a significant simple effect of time within the right VLPFC stimulation and high choice conflict conditions (OR = 1.30, 95% CI [1.01, 1.69], *p* = 0.042; Table S2). Additionally, a *Stimulation Type* × *Time* interaction indicated a significant difference between the right and left VLPFC stimulation conditions (OR = 0.54, 95% CI [0.38, 0.77], *p* = 0.001; Table S1). A similar pattern was observed in the right vs. bilateral comparison, though it did not reach statistical significance (OR = 0.75, 95% CI [0.52, 1.08], *p* = 0.12).

Conversely, left VLPFC stimulation was associated with a significant reduction in LL choices (suggesting a potential increase in impulsivity) manifested as a significant simple effect of Time (OR = 0.71, 95% CI [0.56, 0.91], *p* = 0.006). A similar analysis of bilateral VLPFC stimulation indicated it did not significantly alter intertemporal choices (OR = 0.98, 95% CI [0.76, 1.26], *p* = 0.864).

No significant three-way interaction (*Stimulation Type* × *Time* × *High-Conflict Indicator*) or other two-way interactions were observed. All covariates had significant main effects (*p* < 0.001 for *High-Conflict Indicator, Sum Ratio, SS Amount, Payment Time Difference*; *p* = 0.031 for *Trial Type*), suggesting that results were valid with participants considering reward magnitude and delay when making choices. When controlling for session order, we observed a main effect of session (χ^2^1 = 5.05, *p* = 0.025), with participants generally increasing their LL choices over sessions (OR = 1.16, 95% CI [1.02, 1.32], *p* = 0.025). Crucially, the critical Stimulation Type × Time interaction effect remained significant (χ^2^2 = 11.18, *p* = 0.004) and the general pattern of the results remained consistent, demonstrating that order effects did not account for the observed stimulation effects.

Repeating the analysis after omitting the High-Choice Conflict indicator showed consistent results (Stimulation Type × Time: χ^2^2 = 10.45, *p* = 0.005). Analysis restricted to high-conflict trials also showed a significant effect (χ^2^2 = 12.17, *p* = 0.002) confirming the pattern held across different analytical approaches.

Finally, to verify that results were not driven by individual participants, we systematically excluded each participant and re-ran the model using the leave-one-out paradigm. For the full dataset, the Stimulation Type × Time interaction remained significant in 10 of 11 iterations (*p*-values ranged from < 0.001 to 0.044) and marginally significant in one iteration (*p* = 0.076). When restricting analysis to high-conflict trials only, the interaction was significant across all 11 iterations (p-values ranged from < 0.001 to 0.031), demonstrating complete robustness of the effect in the theoretically critical decision context. These results indicate that while individual participants can influence effect strength with n = 11, the core pattern, particularly for high-conflict choices where self-control is most engaged, is robust to single-participant removal.

### Emotional reactivity task performance

**Fig. 4:**
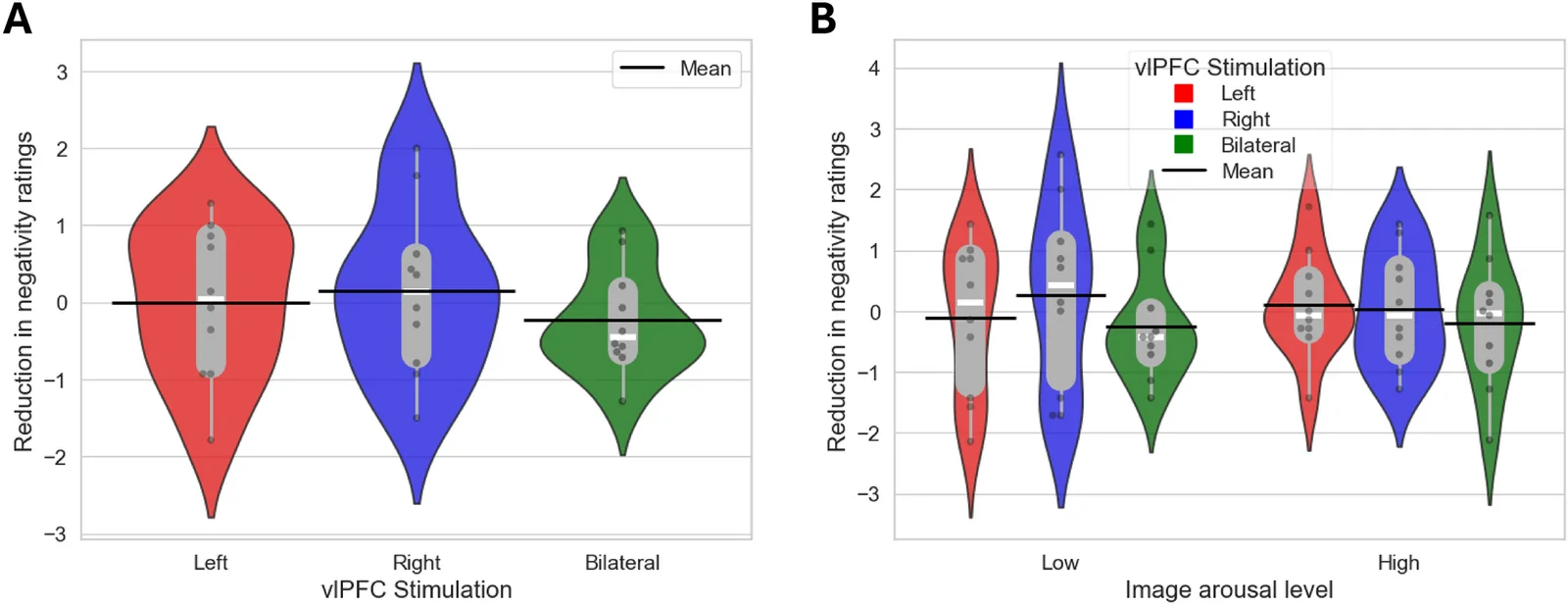
Change in negativity ratings in response to social isolation images following stimulation of different VLPFC targets (pre minus post-stimulation). Higher values indicate a greater reduction in negativity ratings after stimulation. (**A**) Aggregated differences across all images. (**B**) Changes across low- and high-arousal social isolation images. While not statistically significant, numerically, negativity ratings for low-arousal images decreased following Right VLPFC stimulation compared to Left and Bilateral VLPFC stimulation. The latter showed a qualitative (non-significant) increase. The dark dots represent individual participant data points.

We observed a significant main effect of the Image Arousal Category (χ^2^_1_ = 83.82, *p* < 0.001; Table S3), confirming that participants engaged with the task and reported meaningful ratings. Linear-mixed model analysis of emotional negativity rating in response to social isolation images revealed a significant main effect of *Stimulation type* (χ^2^_2_ = 9.30, *p* = 0.010; Table S3). However, we did not find a significant *Stimulation type x Time* interaction (χ^2^_2_ = 1.58, *p* = 0.454)*,* nor a significant *Stimulation type x Time x Image arousal interaction* (χ^2^_2_ = 0.51, *p* = 0.775), indicating that no stimulation type could significantly change emotional negativity rating (Fig. 4A).

To further explore potential effects, we examined stimulation-induced changes specifically within the low-arousal image condition (i.e., set as the reference level). While no significant effects emerged, the data showed a numeric decrease in negativity ratings following right VLPFC stimulation (β = −0.06, 95% CI [−0.18, 0.07], *p* = 0.38; Table S4) and a slight increase following left (β = 0.03, 95% CI [−0.09, 0.16], *p* = 0.629) and bilateral VLPFC stimulation (β = 0.05, 95% CI [−0.07, 0.18], *p* = 0.411) (Fig. 4B ). Re-running the model while controlling for session order did not change the pattern of results with no significant *Stimulation type × Time* interaction (χ^2^_2_ = 1.59, *p* = 0.451)*,* nor a significant *Stimulation type × Time × Image arousal interaction* (χ^2^_2_ = 0.53, *p* = 0.769).

## Discussion

To our knowledge, this is the first study to test the effect of VLPFC-targeted rTMS on the core symptoms of BPD. Our findings indicate that stimulation of the right but not left or bilateral VLPFC-amygdala circuit with a single session of iTBS resulted in a significant reduction in a behavioral measure of impulsivity in BPD. While we found no significant changes in emotional reactivity, the numerical reduction in emotional reactivity warrants its closer examination in a future sufficiently powered study. Based on these findings, we suggest right VLPFC as a promising novel brain circuit-based target for reducing the intensity of impulsivity in individuals with BPD.

Patients with BPD have been shown to consistently prefer immediate discounted rewards to larger delayed rewards in discounting tasks^41^; this is one of the best established neuropsychological tasks for measuring behavioral impulsivity in human^42^. We showed that neuromodulation of the right VLPFC-amygdala circuit can effectively increase self-control to resist sooner but smaller rewards, which could translate to reduced impulsive decision-making in BPD. In contrast, the left VLPFC stimulation appeared to have the opposite effect and increased impulsivity. Interestingly, bilateral VLPFC stimulation did not produce significant changes, possibly due to the opposing effects of right- and left-sided stimulation canceling each other out. A recent study examined the effect of rTMS over the right dorsolateral prefrontal cortex for modulating delay discounting task performance in a group of BPD patients but did not find any significant effect^43^. VLPFC appears to be a more promising target to affect this behavior.

Achieving greater control over impulsivity (e.g., substance abuse, overspending, hypersexuality, reckless driving, or binge eating) is an important therapeutic goal in BPD. Self-mutilation and suicidal behaviors also are significant manifestations of impulsiveness in BPD, and contribute to a rate of completed suicide of 10% (50 times higher than the general population)^44^. Impulsive behaviors are very often closely related to other symptoms of BPD. For example, feelings of emptiness or fear of abandonment may be so disturbing that affected individuals engage in impulsive self-destructive acts to avoid those negative thoughts and emotions. High impulsivity has been shown as a negative predictor for treatment response^45^ and remission in BPD^46^. There is currently no effective pharmacological treatment option for this core feature of BPD^45^.

Our finding of a right- but not left-sided significant effect of stimulation on the target behaviors should be interpreted in the context of neuroimaging studies in BPD. Results of two recent meta-analyses of BPD showed abnormal structural (smaller gray matter volume)^22^ and functional (hypoactivation during emotion processing tasks)^21,22^ of the right (but not left) VLPFC (labeled as IFG in those studies) in BPD compared to healthy controls. These earlier findings are consistent with the present finding that selective stimulation of the right VLPFC results in a reduction in behavioral impulsivity in BPD subjects, potentially through enhanced top-down control of the right amygdala stemming from ventrolateral frontal stimulation.

The numeric trend towards decreased emotional reactivity following right VLPFC stimulation is encouraging, and aligned with previous studies showing that stimulation of this target enhances emotional reappraisal in healthy subjects. Zhang and colleagues showed that right VLPFC stimulation in control subjects enhanced emotion regulation in response to social exclusion via a reappraisal mechanism^47,48^. Another recent fMRI/TMS study found that rTMS over the right VLPFC in healthy controls reduced amygdala activity during emotion regulation via cognitive reappraisal startegy^49^. Anodal transcranial Direct Current Stimulation (tDCS) of the right VLPFC also was shown to enhance inhibitory control (response inhibition)^50,51^. The present findings extend this earlier work in healthy populations and suggest that the right-sided VLPFC-amygdala circuit could constitute a novel stimulation target to effectively enhance frontolimbic top-down regulatory processes in clinical populations as well. It is possible that a more significant effects on emotional reactivity might have been observed if we had tested the effect on emotion regulation specifically via cognitive reappraisal, considering previous work and the well-known role of the VLPFC in reappraisal. Alternatively, it is possible that more than a single rTMS session is needed to induce a meaningful change in emotional reactivity. Both of these possibilities should be examined in future studies of the potential enhancement of control over emotional regulation in BPD.

The innovative strengths of this study include use of neuroimaging guidance to identify VLPFC targets for each participant, in contrast to previous rTMS studies in BPD that have used more traditional depression targets such as the dorsolateral or dorsomedial prefrontal cortex and targeting with scalp-based methods^52,53^. The contrast of differential results from left, right, and bilateral stimulation is uniquely informative regarding the effects of VLPFC stimulation in BPD.

These strengths are tempered by several limitations that warrant cautious interpretation of the findings. First, the modest sample size constrains participant-level inference and may inflate effect size estimates and increase susceptibility to Type I error, despite the increased sensitivity afforded by the within-subject crossover design in which each participant serves as their own control. To address concerns that the observed hemispheric dissociation might be driven by individual participants, we conducted leave-one-out sensitivity analyses, which showed that the critical Stimulation Type × Time interaction was generally preserved across iterations, particularly for high-conflict trials. Nonetheless, given the small sample size, these analyses cannot fully rule out the influence of individual variability on effect estimates, and replication in larger, well-powered samples will be essential to confirm the robustness of the effects we found. Second, we examined only one measure to quantify impulsivity (delayed discounting). While this is one the most established neurobehavioral tasks for quantifying impulsivity, future studies should consider examining impulsivity with the Go/No-Go, Stop Signal, Iowa Gambling, Continuous Performance Task, or the Cambridge Gambling^54^ and potentially other tasks. Third, we should clarify that we currently do not have evidence to support that a single-session VLPFC stimulation can significantly alter emotional reactivity task performance and therefore, we do not have any strong claim or particular emphasis on the reported numerical trend per se. However, the anatomical location (the right but not left or VLPFC) where that change in reducing emotional reactivity measure was detected is of note and aligned not only with our significant findings that right VLPFC stimulation modulated delayed discounting for the same subjects but also with the previous findings discussed above that right VLPFC rTMS modulates emotion regulation in healthy subjects. Finally, our emotional regulation task mainly addresses the dimension of reactivity to social exclusion, and other measures of emotional regulation should be considered.

This randomized crossover trial comparing right-, left-, and bilateral VLPFC-amygdala circuit rTMS indicates that individualized, imaging-based stimulation of the right VLPFC can be used to safely and effectively modulate core behavioral measures of impulsivity and possibly emotional reactivity in subjects with BPD. These findings are encouraging and warrant exploration of this target circuit through clinical trials involving extended rTMS treatment over multiple sessions. Such trials could determine if VLPFC-amygdala rTMS might be used to ameliorate core BPD symptoms and provide enduring clinical benefit.

## Supplementary Information

Supplementary Information.

## Data Availability

The datasets generated during and/or analyzed during the current study are available from the corresponding author on reasonable request.
