# Supplementary material for: Ventrolateral prefrontal-amygdala repetitive transcranial magnetic stimulation (rTMS) modulation of impulsivity in borderline personality disorder: a proof-of-concept study
Source: Sci Rep. 2026 Jun 17;16:26833. doi: 10.1038/s41598-026-56692-2 (PMC13518863; doi:10.1038/s41598-026-56692-2)
Supplement: Supplementary file 1 [file 41598_2026_56692_MOESM1_ESM.docx]

**Supplementary Materials**

**Ventrolateral prefrontal-amygdala Repetitive Transcranial Magnetic Stimulation (rTMS) modulation of impulsivity in Borderline Personality Disorder: A proof-of-concept study**

Reza Tadayonnejad, M.D., Ph.D.**^ǂ,*^**^,1,2,3^, Rani Gera, Ph.D.**^ǂ^**^,3^, Stephanie A. Chu, B.S.^1,2^, Hope Hawkins, B.S. ^1,2^, Emmily Hovhannisyan, B.A.^1,2^, Thuc Doan P. Ngo, B.A.^1,2^, Juliana Corlier, Ph.D.^1,2^, Andrew F. Leuchter, M.D.^1,2^

**RESULTS**

***Supplementary tables***

**Type II Wald Chi-Square Tests for the Mixed-Effects Logistic Regression Model Predicting Intertemporal Choice Behavior.**

**Logistic Regression Model Predicting Intertemporal Choice Behavior. Right vlPFC stimulation and High choice conflict were used as baseline levels**

**Type II Wald Chi-Square Tests for the Mixed-Effects Linear Regression Model Predicting Negativty ratings in response to social isolation images..**

**Linear Regression Model Predicting Negativty ratings in response to social isolation images. Right vlPFC stimulation and Low arousal image category used as baseline levels.**
